# Geometric close-packing mechanism for predicting short-range correlations in nuclei

**DOI:** 10.1038/s41598-026-45765-x

**Published:** 2026-04-08

**Authors:** Jingfeng Lin

**Affiliations:** South China University of Technology, Guangzhou, 510641 China

**Keywords:** Biophysics, Physics

## Abstract

A geometric close-packing framework for nuclear structure is introduced, delivering parameter-free predictions for neutron–proton (*np*) short-range correlation (SRC) pair counts across the nuclear chart. Motivated by QCD-inspired parton flux tubes and electromagnetic resonance phenomena observed in BESIII measurements, each nucleon is represented by a closed double-helix parton flux tube configuration following a toroidal trefoil-knot trajectory and assembling into concentric toroidal shells sharing a common centroid. Within this framework, SRCs arise naturally as nearest-neighbor contacts between adjacent flux tube segments, converting SRC counting into a deterministic consequence of spatial organization rather than a phenomenological input. The resulting *np*-SRC counts quantitatively reproduce CLAS measurements for C, Al, Fe, and Pb and are consistent with established experimental systematics. The geometry further indicates a set of correlated features of *np*-SRC dynamics: (i) each nucleon participates in at least one *np*-SRC pair; (ii) adding neutrons increases the fraction of high-momentum protons; and (iii) adding protons increases the fraction of high-momentum neutrons. These results suggest that short-range nuclear dynamics is governed by an underlying geometric organizing principle, offering a physically transparent mechanism for the emergence of SRCs in nuclei.

## Introduction

The study of short-range correlations (SRCs) between nucleons dates back to the 1950s^1,2^, long before the discovery of the EMC effect^3,4^. Over the past decades, extensive experimental efforts have established SRCs as a universal feature of nuclei. Measurements using electron scattering, hadron-induced reactions^5^, and photon probes^6^ consistently show that SRCs are dominated by neutron–proton (*np*) pairs carrying high relative momentum but small center-of-mass (c.m.) momentum^1,7^. A recent review^1^ summarizes the growing body of evidence for the universality of *np*-SRC pairs across nuclei and experimental probes. The Jefferson Lab E08-014 experiment^8^ ($\textit{A}(e,{e}^{'})$), extending the earlier measurement^9^ to broader kinematic coverage and additional nuclei, reports inclusive per-nucleus cross-section ratios that reconfirm a key trend: adding neutrons to $^{40}\textrm{Ca}$ increases the number of correlated pairs.

Complementary evidence has been obtained in the CLAS (CEBAF Large Acceptance Spectrometer) in Hall B at Jefferson Lab. Using a 5.014 GeV electron beam, the experiment measured both proton and neutron knockout reactions with high signal purity and reduced background contributions^10^. By simultaneously detecting $(e,{e}^{'}p)$ and $(e,{e}^{'}n)$ events, the experiment directly compared the high-momentum proton and neutron components within the same nuclei. The use of $A/\text {C}$ double ratios—defined as the ratio of high-momentum (SRC) to low-momentum (mean-field) nucleon yields in nucleus *A*, normalized to the same ratio in carbon— further suppressed systematic uncertainties by canceling normalization, transparency, and detection-efficiency effects. For the CLAS measurements, *A* denotes nuclei such as Al, Fe, and Pb, and the double ratio serves as an estimator of the relative enhancement of SRC nucleons in asymmetric nuclei compared to carbon. These measurements demonstrated that the neutron excess in neutron-rich nuclei increases the fraction of high-momentum protons, providing strong evidence that *np*-SRC pairs remain the dominant source of high-momentum nucleons even in heavy nuclei^11,12^. Despite this progress, the structural mechanism by which excess neutrons generate such close-proximity *np* pairs remains incompletely understood.

Most theoretical descriptions treat SRCs using effective contact interactions^13,14^, correlation operators, or phenomenological scaling relations. While these approaches reproduce many observables, they typically encode correlations in momentum space and leave open a complementary question: what spatial organization of nucleons in position space^6^ renders SRC formation inevitable across nuclei?

The present work addresses this question from a geometric perspective. Building on QCD-inspired flux tube concepts^15^, hadron–hadron scattering amplitudes^16–21^, and electromagnetic resonance phenomena observed in BESIII measurements^22^, each nucleon is represented by a closed parton flux tube configuration following a toroidal trefoil-knot trajectory. In the nuclear core, close packing of such extended nucleons into hexagonal lattice arrangements^23^ reveals a previously unreported nucleon-number regularity: the nucleon numbers of the most abundant isotopes approximately follow $S(S+1)-2$, while neutron numbers in fully filled configurations coincide with known magic numbers (2, 8, 20, 28, 50, 82, 126^24^, and 108^25^).

Within this framework, geometric close packing naturally generates nearest-neighbor *np* contacts throughout the dense nuclear core. Each nucleon participates in at least one such contact, providing a structural correspondence to the experimentally observed dominance of *np*-SRC pairs. Because nucleons are arranged around a common center of mass within the lattice configuration, the resulting pairs are consistent with the near-zero c.m. momentum observed experimentally. Combinatorial counting of nearest-neighbor *np* contacts based on proton and neutron numbers reproduces several trends observed in CLAS measurements, including the relative fractions of high-momentum nucleons, the characteristic dependence on (*N*/*Z*), and the saturation behavior of SRC scaling factors^10–12^.

The present study therefore links nucleon-number geometric systematics directly to experimentally measurable SRC signatures. This framework describes ground-state nuclear structure independent of specific reaction mechanisms or momentum-transfer conditions. It thus provides a structural perspective that may assist the interpretation of ongoing and planned high-precision SRC measurements^1^, including experimental programs involving $^{40}\textrm{Ca}$–$^{48}\textrm{Ca}$–$^{54}\textrm{Fe}$ isotopes^40,44^.

## Nucleon structure and geometric packing

**Fig. 1:**
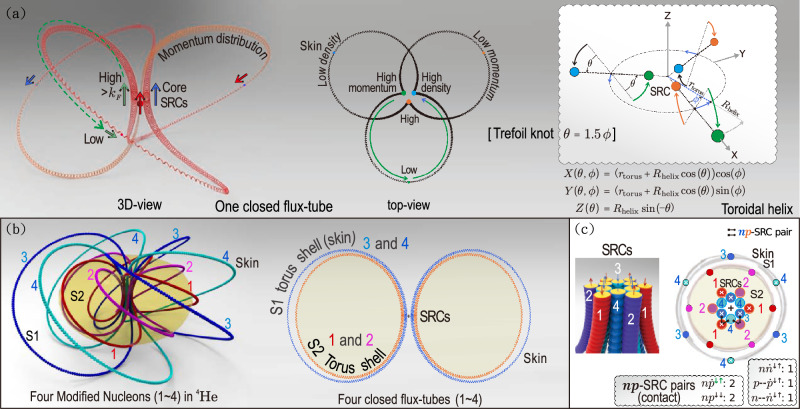
Geometric structure of nucleons and formation of SRCs. **a** Each nucleon is represented by a closed double-helix parton flux tube following a toroidal trefoil-knot trajectory, yielding a spatially extended structure. The inset and formula illustrate topological combination: double helix + torus^26^ + trefoil knot^27–29^. **b** Arrangement of four closed flux tubes in $^{4}\textrm{He}$, illustrating the nucleon binding configuration. Two concentric toroidal shells (S1, S2) are depicted, and SRCs emerge within the central high-density flux tube cluster. **c** Transverse cross-section of the dense, close-packed nuclear core in $^{4}\textrm{He}$. Red and blue circles denote the transverse cross-sections of proton and neutron flux tubes, respectively. The outlined box lists the counts of several nucleon-nucleon pair types and key markings: $\mathrm{NN}$ (nucleon-nucleon) denotes SRC contact pairs; $\mathrm{\dot{N}}$ denotes that its flux tube current orientation is opposite to that of its partner; $\mathrm{N--\dot{N}}$ denotes zero-spin pairs symmetric about the central point $\mathrm{\dot{N}}$. Symbols $\otimes$ and $\Circle$ denote the direction of the internal flux tube circulation projected along the axis normal to the cross-section, pointing into and out of the page, respectively.

Motivated by QCD-inspired flux tube structures^15^ and by electromagnetic resonance phenomena observed in BESIII experiments^22^, each nucleon is represented by a closed double-helix parton flux tube configuration following a toroidal trefoil-knot trajectory, as illustrated in Fig. 1a. Within a nucleus, these spatially extended nucleons assemble into a dense central configuration forming a high-density nuclear core, which serves as the primary locus for the formation of short-range correlations (SRCs), as illustrated in Fig. 1b–c.

All nucleons share a common centroid and are arranged through their parton flux tubes in concentric toroidal shells, forming a nested toroidal structure (Fig. 1a). Within this framework, the spatially extended nucleons participate in interactions within a dense nuclear core where center-of-mass momenta remain comparatively small^30^. Transverse oscillations of the flux tubes periodically bring partons into short-range proximity, generating high-momentum components consistent with the universal short-distance dynamics observed across nuclei^10^. This three-dimensional assembly forms a central high-density flux tube cluster (Fig. 1c) and produces a close-packed configuration.

**Fig. 2:**
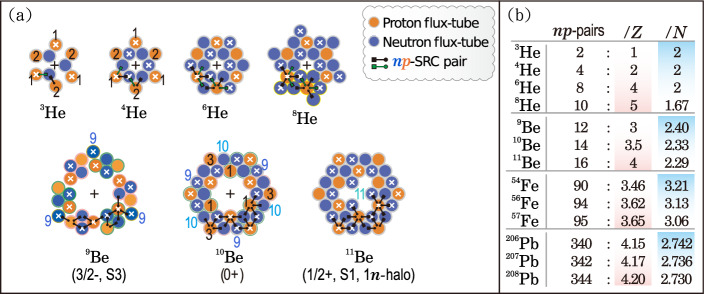
From arrangement rules to *np*-SRC pair counts. **a** Example SRC arrangements for four helium and three beryllium isotopes, illustrated via core cross-sections. Red and blue circles denote transverse sections of proton and neutron flux tubes, respectively. The arrangement rules follow from nucleon number geometric systematics (NNGS)^23^, in which every nucleon participates in at least one *np*-SRC pair. odd-indexed toroidal shells tend toward full neutron occupation, linking geometric packing to the emergence of magic numbers. Shell S1 is both the largest toroidal shell (outermost in three-dimensional) and closest to the centroid in cross-section. S1 corresponds to the lowest-energy orbital. **b** Total *np*-SRC pair counts for 13 representative isotopes of He, Be, Fe, and Pb. The counts show that the number of *np*-SRC pairs per proton increases with neutron number, while the corresponding number per neutron decreases slightly. SRC arrangements for Fe and Pb isotopes are provided in Extended Data Figs. 6 and 7.

In transverse core cross-sections (Fig. 1c), the arrangement naturally forms a concentric hexagonal pattern that can dynamically reorganize following excitation or nucleon removal, while preserving the preferred nearest-neighbor connectivity of the lattice. Each site can form up to six nearest-neighbor contacts (Fig. 2a). These contacts define the geometric sites of SRC formation, making SRCs a direct consequence of flux tube close packing in real space. Within this framework, SRCs arise from geometric constraints governing how extended nucleons assemble at high density, rather than from fitted phenomenological assumptions.

Notably, building on earlier analysis of nucleon-number geometric systematics (NNGS)^23^, 37.2% of the most abundant even-*Z* isotopes lie on or close to the relation $A = S(S+1)-2$ (with *S* the shell index), corresponding to nearly complete triple-symmetric hexagonal arrays (see Extended Data Fig. 7). Representative nuclei with nucleon numbers $A \simeq S(S+1)-2$ include $^4$He, $^{12}$C, $^{16}$O, $^{20}$Ne, $^{40}$Ca, $^{52}$Cr, $^{56}$Fe, $^{69,71}$Ga, $^{88}$Sr, $^{90}$Zr, $^{106}$Pd, $^{107,109}$Ag, $^{130}$Te, $^{132}$Xe, $^{152}$Sm, $^{153}$Eu, $^{180}$Hf, $^{208}$Pb, and $^{238}$U, as well as the unstable doubly magic nucleus $^{270}$Hs^25^. The same nucleon-number regularity also appears in unstable elements such as technetium and promethium, whose nearest stable neighbors follow the same geometric sequence (Extended Data Fig. 6). Moreover, several elements exhibiting atomic-weight inversion in the periodic table—including $^{40}$Ar, $^{130}$Te, and $^{238}$U—have mass numbers close to the geometric shell condition $A = S(S+1)-2$, providing empirical support for the proposed nucleon-number systematics.

Although one might expect the organizational principles of nucleons to differ between light and heavy nuclei, inclusive electron scattering reveals universal high-momentum tails across the nuclear chart^31,32^, while the EMC slope rises monotonically with mass number *A* in all measured nuclei. Both observations point to a common underlying organization governing nuclear short-range dynamics. Within this framework, nucleon packing is governed by a small set of core geometric packing rules:

1. Universal *np* participation: Guided by the nucleon-number systematics^23^, every nucleon must participate in at least one *np*-SRC pair. This condition is satisfied by close packing and is essential for stability.
2. Neutron-favored filling of odd-indexed shells: In the core cross-sectional geometry, neutrons in an odd-indexed shell naturally arrange along the edges of a hexagonal ring, forming a closed neutron loop. Adjacent neutrons adopt alternating orientations of the internal flux tube circulation, corresponding to opposite directions of the flux tube current projected along the axis normal to the cross-section. The symbols used to denote these orientations are defined in Fig. 1c. Such a configuration enables each neutron in the loop to form nearest-neighbor neutron–proton pairing with one or more protons residing in the adjacent even-indexed shells. Notably, cumulative nucleon numbers for successive fully occupied odd-indexed shells align spatially with empirical magic numbers (see Figs. 5–7 and Refs.^23,24^). In this sense, this geometric constraint system is compatible with, and spatially maps onto, the observed shell closures. Furthermore, the closed neutron-loop geometry is consistent with experimental indications of quasi-bound neutron–neutron correlations^33^.
3. Proton-packing constraint: All protons share the same internal structure and differ only by an overall spatial orientation. In a multi-nucleon system, directly neighboring protons are constrained to adopt opposite orientations, corresponding to antiparallel parton current configurations at contact. Such antiparallel currents generate an effective Ampère-type transverse repulsion, preventing protons from approaching arbitrarily closely and enforcing a minimum separation. By contrast, parallel current configurations lead to enhanced attractive interactions and therefore cannot form stable nearest-neighbor proton–proton contacts. As a result, parallel protons must be separated by neutrons. Neutrons thus play a dual role: while mediating the spatial separation of parallel proton configurations, all neighboring neutron–proton pairs remain continuously coupled. Within this model, this persistent coupling manifests as an electromagnetic-like orthogonal oscillatory interaction, consistent with the orthogonal proton–neutron oscillations observed in BESIII measurements^22^.
4. Spin-pairing tendency: Identical nucleons in the same shell preferentially form rotationally symmetric spin-zero pairs, consistent with Pauli exclusion and observed pairing phenomena.
5. Outer-shell geometry: The outermost shell governs neutron skins, proton skins, and $\alpha$-like clustering, linking SRC geometry to surface phenomena.

With the close-packing rules established, SRC pairs are identified with nearest-neighbor contacts in the dense core. No additional SRC probability or strength is introduced. The total numbers of *np*-SRC, *pp*-SRC, and *nn*-SRC pairs follow directly from the geometry (see Figs. 5–7).

## Comparison with experimental systematics

**Fig. 3:**
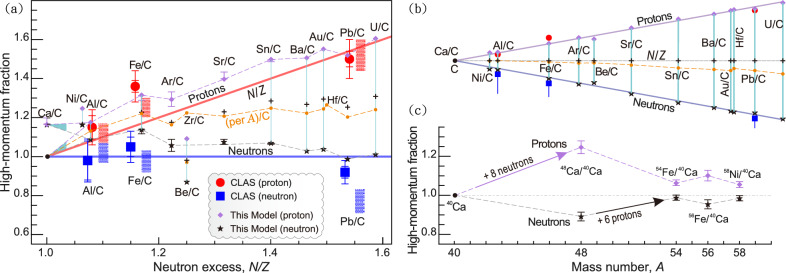
Systematics of predicted *np*-SRC pair counts and comparison with experimental data. **a** CLAS data^10^ (Table 2): Red circles with error bars denote the double ratio of the number of $(e,e'p)$ high-momentum proton events to low-momentum proton events for nucleus *A* relative to carbon. Blue squares with error bars show the same for neutron events. Red and blue rectangles show the range of predictions of the phenomenological *np*-dominance model. This work: Purple diamonds (protons) and black stars (neutrons) show predictions obtained from geometric *np*-SRC pair counting (Table 1). Note that *N* and *Z* correspond to their low-momentum event counts, respectively (see Ref.^10^ for details). The orange line represents the per-nucleon *np*-SRC pair ratio, normalized to $^{12}$C. The $^{12}$C reference value is taken as 16.5 to account for experimental uncertainties, and Pb results reflect natural isotopic abundances. Cyan vertical lines denote the spacing lines. **b** Transformed curve plot with spacing lines aligned at their central points, comparing the geometric combinatorial trend predicted by this model with experimental systematics plotted versus *N*/*Z*. Deviations for nuclei such as $^{58}\textrm{Ni}$ and $^{9}\textrm{Be}$ indicate that the trend is not determined by *N*/*Z* alone. **c** Predictions of the high-momentum proton (neutron) fractions relevant to planned and ongoing $^{40}$Ca–$^{48}$Ca–$^{54}$Fe experiments^40,44^.

Guided by the nucleon-number geometric systematics^23^, this model yields a strong dominance of neutron–proton (*np*) short-range correlated pairs^34,35^, as well as the observed saturation of the SRC scaling factor $a_2(A/\textrm{d})$ in medium-to-heavy nuclei—consistent with experimental observations. Experimentally, $a_2(A/\textrm{d})$ is commonly interpreted as a measure of the number of deuteron-like *np*-SRC pairs in nucleus *A* relative to deuterium^14^. In the present framework, this saturation arises directly from geometric saturation of nearest-neighbor contacts in the dense nuclear core, reflecting how structural packing constraints translate to observable SRC signatures.

A central structural consequence of the close-packing geometry is that every nucleon participates in at least one *np*-SRC pair. As additional nucleons are added, geometric constraints favor configurations in which nucleons—particularly minority species—form multiple *np* nearest-neighbor contacts whenever possible. As a result, increasing the neutron-to-proton ratio enhances the number of *np* contacts involving protons, while the average number of *np* contacts per neutron is correspondingly diluted. In proton-richer isotones (e.g., $^{58}$Ni relative to $^{56}$Fe or $^{54}$Fe relative to $^{48}$Ca), the high-momentum neutron fraction is enhanced.

The observed *N*/*Z* asymmetry in SRC participation emerges naturally from real-space combinatorics and packing constraints, without the introduction of adjustable parameters or isospin-dependent tuning. By making the geometry explicit, applying the geometric rules to representative nuclei from $^4\text {He}$ to $^{238}\text {U}$ yields explicit predictions for *np*-SRC pair counts with no free parameters (see Fig. 3a).

**Table 1:** Predicted counts of *np* short-range correlated (SRC) pairs for representative nuclei. Within this geometric close-packing framework, SRC observables are determined once the packing rules are set (Figs. 1, 2 and Extended Data Figs. 5–7). Values in parentheses denote alternative geometric configurations allowed by the packing rules; distinct structures (e.g., neutron-skin^36^ or $\alpha$-skin geometries) are compatible with nuclear shape coexistence^37^.

Nucleus	$\alpha$ skin	*n* skin	per *p*	per *n*	per *A*	*N*/*Z*
$^4$He	–	4	2.00	2.00	1.00	1.00
$^9$Be	12	(12)	3.00	2.40	1.33	1.25
$^{12}$C	(18)	16	2.67	2.67	1.33	1.00
$^{16}$O	22	–	2.75	2.75	1.38	1.00
$^{27}$Al	43	41	3.23	3.00	1.56	1.08
$^{40}$Ar	66 (62)	(60)	3.67	3.00	1.65	1.22
$^{40}$Ca	66	(64)	3.30	3.30	1.65	1.00
$^{48}$Ca	82	80^38^	4.00	2.86	1.67	1.40
$^{56}$Fe	94	(92)	3.62	3.13	1.68	1.15
$^{58}$Ni	96	–	3.43	3.20	1.66	1.07
$^{88}$Sr	146	–	3.84	2.92	1.66	1.32
$^{120}$Sn	204^36^	204^36^	4.12	2.94	1.72	1.40
$^{138}$Ba	–	232	4.14	2.83	1.68	1.46
$^{180}$Hf	310	–	4.31	2.87	1.72	1.50
$^{197}$Au	336	–	4.27	2.86	1.71	1.49
$^{208}$Pb	(344)	344^38^	4.20	2.73	1.65	1.54
$^{238}$U	406	–	4.41	2.78	1.71	1.59

In Fig. 3b, spacing lines are aligned at their central points, revealing a systematic increase in spacing with *N*/*Z*—a feature that reflects the combinatorial trends inherent to this model. Figure 3c presents predictions for high-momentum proton (neutron) fractions, which are directly relevant to planned and ongoing experimental campaigns involving $^{40}\text {Ca}$–$^{48}\text {Ca}$–$^{54}\text {Fe}$ isotopes. These model-based insights provide a coherent framework for understanding the dominance of *np*-SRC pairs, their enhancement in neutron-rich nuclei, and the universal systematics of SRCs across the nuclear chart.

## Uncertainties in *np*-SRC pair counting

While the *np*-SRC pair counting is parameter-free, theoretical uncertainties arise from ambiguities in the specific spatial arrangement of nucleons, with magnitudes constrained by this model’s core assumptions. These uncertainties are small and well-bounded, as detailed below:

For nuclei with uniquely defined packing configurations—including $^4\text {He}$, $^{16}\text {O}$, $^{54}\text {Fe}$, $^{58}\text {Ni}$, $^{88}\text {Sr}$, $^{138}\text {Ba}$, $^{180}\text {Hf}$, and $^{238}\text {U}$—the *np*-SRC pair count is uniquely fixed by the packing rules. For $^4\text {He}$, this results in an unambiguous *np*-pair count of 4, corresponding to the “simple pair counting” scenario described in Refs.^9,32^.

In contrast, some nuclei admit multiple packing arrangements consistent with the geometric constraints, leading to a small spread in *np*-pair count predictions. Representative examples include $^{12}\text {C}$, $^{27}\text {Al}$, $^{40}\text {Ar}$, $^{40}\text {Ca}$, $^{48}\text {Ca}$, $^{56}\text {Fe}$, $^{120}\text {Sn}$, and $^{208}\text {Pb}$, for which both neutron-skin and alpha-skin (S2 shell occupied by $^4\text {He}$-like clusters) configurations are viable. This ambiguity aligns with experimental and theoretical studies of tin isotopes, which report a tight interplay between $\alpha$-cluster formation and neutron-skin thickness^36^, as well as neutron-skin variations along isotope chains. For $^{120}\text {Sn}$ and $^{208}\text {Pb}$, the *np*-pair count is identical across competing configurations. For $^{48}\text {Ca}$, the predicted *np*-pair counts are 80 and 82, corresponding to a maximal variation of $\pm 1.25\%$. The largest uncertainty in the present model arises for $^{40}\text {Ar}$, which admits three configurations with 62, 64, and 66 *np* pairs—yielding a maximal variation of $\pm 3.1\%$. This is smaller than the CLAS experimental uncertainties ($\pm 5.8$–$10\%$) and narrower than the spread of the phenomenological *np*-dominance model ($\pm4.0$–$7.8\%$).

Notably, this model’s allowance for multiple geometric arrangements is compatible with shape coexistence phenomena^37^ observed in many nuclei. Distinct nucleon configurations—each satisfying the close-packing rules but differing in key details of nucleon distribution (e.g., neutron-skin or alpha-skin characteristics)—correspond to distinct nuclear eigenstates. These states might manifest experimentally through measurable variations in quadrupole moments and transition rates, though definitive confirmation of this correspondence awaits future systematic experimental investigations.

## Summary and discussion

Quantifying neutron–proton short-range correlated (*np*-SRC) pairs remains a central challenge in nuclear physics. Guided by nucleon-number geometric systematics, this work proposes a combinatorial mechanism for nucleon close packing in the high-density nuclear core, enabling predictions for the number of *np*-SRC pairs in nuclei ranging from $^3\text {He}$ to $^{238}\text {U}$.

An experimentally testable conclusion of this framework is that the addition of protons enhances the fraction of high-momentum neutrons in nuclei. Within the geometric close-packing picture, this effect follows directly from the dominance of *np*-SRC pairs. The prediction can be tested through species-dependent SRC ratio observables in CLAS measurements, providing a clear experimental benchmark for the proposed mechanism.

Furthermore, the EMC effect—observed as a modification of deep-inelastic scattering ratios in $\textit{A}(e,e')$ reactions relative to deuterium (e.g., SLAC E139, 8–20 GeV)—is empirically correlated with SRCs^39^. SRCs may influence the internal structure of bound nucleons, for example through modifications of parton distribution functions, providing an important physical perspective on the origin of the EMC effect.

**Fig. 4:**
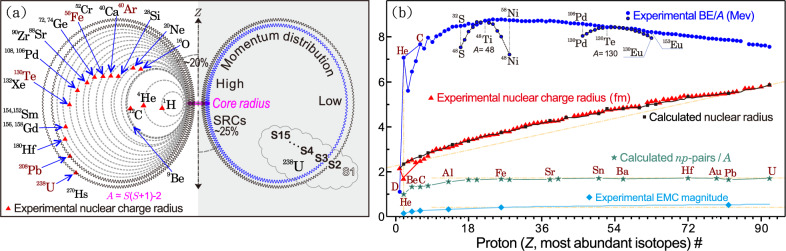
Nucleon expansion, nuclear radius, and the EMC effect. **a** Schematic of the nuclear charge-radius evolution for dominant isotopes corresponding to 15 shell indices. Nucleon expansion within the nucleus is illustrated using $^{238}$U. The 20–25%^13,40^ radial fraction represents the spatial probability region associated with high-momentum parton components contributing to SRC dynamics in the dense nuclear core. **b** Green stars denote the predicted per-nucleon *np*-SRC pair counts, following a trend similar to the binding energy per nucleon (BE/*A*, blue circles; data points for non-dominant isotopes at *A*=48 and 130 are marked by black circles–*A* and *N* are not continuous). In contrast, the EMC slope exhibits a monotonic increase (cyan diamonds: experimentally extracted EMC slopes). Black squares show model-calculated nuclear radii (Extended Data Table 3), which scale linearly with measured charge radii (red triangles, Ref.^41^). Yellow guide lines aid in visualizing the trend and curvature of each curve.

Notably, the SRC scaling factor $a_2$ saturates in medium-to-heavy nuclei^39^, whereas the slope of the EMC effect continues to increase with nuclear size. Within the present geometric framework, nucleons embedded in nuclei may exhibit an effective spatial expansion relative to free nucleons (Fig. 4a). Such collective expansion would influence the nuclear radius and may correlate with the magnitude of the EMC effect observed in deep-inelastic scattering (Fig. 4b).

This model further suggests a linear correlation between the size of the dense nuclear core and nuclear radii, supporting the hypothesis that nuclear size may reflect the combined effects of nucleon close packing in SRC-dominated cores and radial nucleon expansion (Fig. 4b, Extended Data Table 3 and Figs. 5–7).

## Supplementary Information

Supplementary Information.

## Data Availability

Data are included in the main text and Extended Data of this paper. Additional raw or processed data that underpin the results are available from the author upon request.
