# Supplementary material for: Geometric close-packing mechanism for predicting short-range correlations in nuclei
Source: Sci Rep. 2026 Apr 8;16:16658. doi: 10.1038/s41598-026-45765-x (PMC13219631; doi:10.1038/s41598-026-45765-x)
Supplement: Supplementary file 1 — Supplementary Information. [file 41598_2026_45765_MOESM1_ESM.pdf]

## Supplementary data

Tables 2 to 3
Figures 5 to 7

**Table 2. Extended Data:** Experimental and theoretical double ratios of high-momentum nucleon fractions (Fig. 3), normalized to $^{12}$C. Column 3 lists CLAS results[10]; Column 4 shows the *np*-dominance model range; Column 5 lists predictions from this work, note that $N$ and $Z$ correspond to their low-momentum event counts, respectively (see Ref.[10] for details); values in parentheses denote alternative packing arrangements (see $^{56}$Fe in Extended Data Fig. 6a and Fig. 7).

| | | Measured ratio | *np*-dominance model | This model |
|---|---|---|---|---|
| Protons | Al/C | $1.15 \pm 0.09$ | 1.06 - 1.17 | 1.18 |
| | Fe/C | $1.36 \pm 0.08$ | 1.20 - 1.30 | 1.32(1.29) |
| | Pb/C | $1.50 \pm 0.10$ | 1.44 - 1.60 | 1.52 |
| Neutrons | Al/C | $0.99 \pm 0.10$ | 0.97 - 1.07 | 1.09 |
| | Fe/C | $1.05 \pm 0.08$ | 0.92 - 1.03 | 1.14(1.12) |
| | Pb/C | $0.92 \pm 0.06$ | 0.71 - 0.83 | 0.99 |

**Table 3. Extended Data: Geometrically derived core radii and nuclear radii for 18 representative nuclei.** The central-cluster (core) radius is directly obtained from the *np*-SRC configurations under the geometric assumption that flux tubes have a circular cross-section of unit diameter (see Extended Data Fig. 5b). The physical size of the core is not determined in the present framework. The nuclear radius is calculated using the empirical relation $R_{nuc} = R_{core}/3.75 + 1.747$ (units: fm), which reproduces the overall trend of the experimental rms nuclear charge radii[41].

| Nuclide | Shell index | Core Radius | Nuclear Radius (cal.) | Charge Radius (exp., fm) |
|---|---|---|---|---|
| $^{4}$He | **1.5** | $\sim$2.23 | $\approx$ 2.34 | 1.676 |
| $^{12}$C | 3 | $\sim$3.50 | $\approx$ 2.68 | 2.470 |
| $^{16}$O | 4 | $\sim$4.11 | $\approx$ 2.84 | 2.699 |
| $^{40}$Ar, $^{40}$Ca | 6 | $\sim$6.07 | $\approx$ 3.36 | 3.427 |
| $^{56}$Fe | 7 | $\sim$7.50 | $\approx$ 3.75 | 3.738 |
| $^{72}$Ge | 8+ | $\sim$8.44 | $\approx$ 4.00 | 4.074 |
| $^{88}$Sr | 9 | $\sim$9.50 | $\approx$ 4.28 | 4.224 |
| $^{98}$Mo | **9.5** | $\sim$10.04 | $\approx$ 4.42 | 4.409 |
| $^{120}$Sn | 11- | $\sim$10.65 | $\approx$ 4.59 | 4.652 |
| $^{130}$Te | 11 | $\sim$11.50 | $\approx$ 4.81 | 4.742 |
| $^{138}$Ba | 11+ | $\sim$11.50 | $\approx$ 4.81 | 4.838 |
| $^{142}$Nd | **11.5** | $\sim$11.63 | $\approx$ 4.85 | 4.912 |
| $^{152}$Sm | 12- | $\sim$12.03 | $\approx$ 4.96 | 5.082 |
| $^{158}$Gd | 12+ | $\sim$12.50 | $\approx$ 5.01 | 5.157 |
| $^{180}$Hf | 13 | $\sim$13.50 | $\approx$ 5.35 | 5.347 |
| $^{208}$Pb | 14 | $\sim$14.06 | $\approx$ 5.49 | 5.501 |
| $^{238}$U | 15 | $\sim$15.50 | $\approx$ 5.87 | 5.857 |

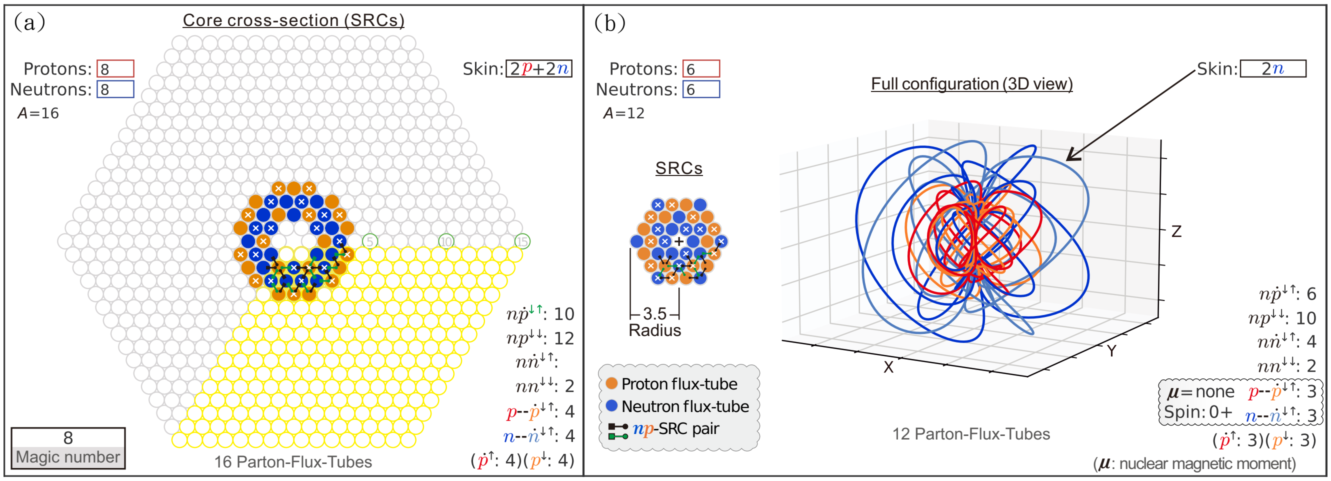


**Figure 5. Extended Data: Computer-assisted analysis of the flux tube structure and counts of various nucleon-nucleon pair types.** (**a**) Identified SRC arrangement and corresponding pair counts in $^{16}$O. (**b**) Three-dimensional flux tube structure in $^{12}$C, showing the neutron skin (S1 shell) and the central high-density flux tube cluster. An alternative configuration of $^{12}$C is shown in Fig. 7. All annotations and symbols are defined in Fig. 1(c).

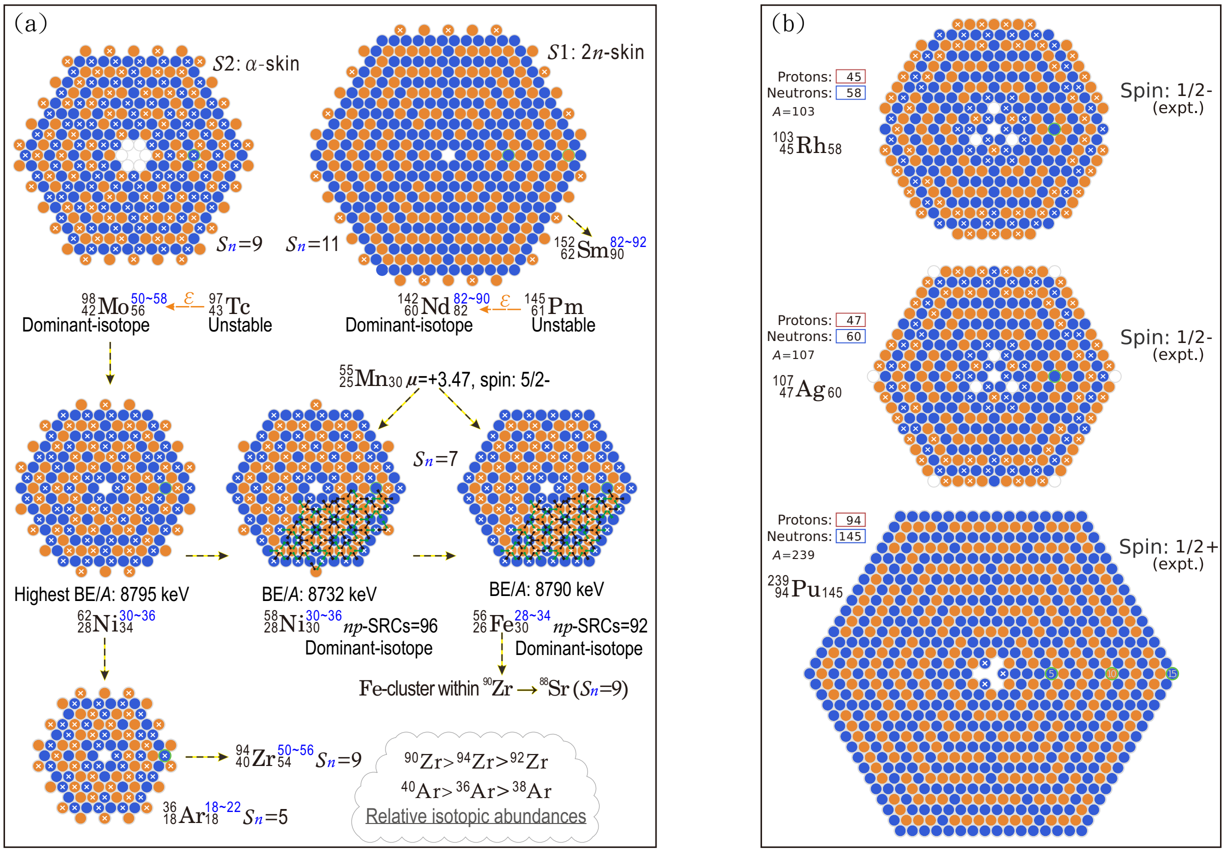


**Figure 6. Extended Data: Consistency of nucleon arrangement configurations with shell index.** **(a)** Similar configurations across representative stable nuclides. An alternative configuration of $^{56}$Fe is shown in Fig. 7. **(b)** Unpaired nucleons in three representative stable nuclides preferentially occupy the S1 shell (low-energy shell) due to symmetry and other ordering rules, consistent with experimental data (spin and magnetic-moment values; see Refs.[42,43] for details). This represents the first nuclear structure model to exhibit this feature[42].

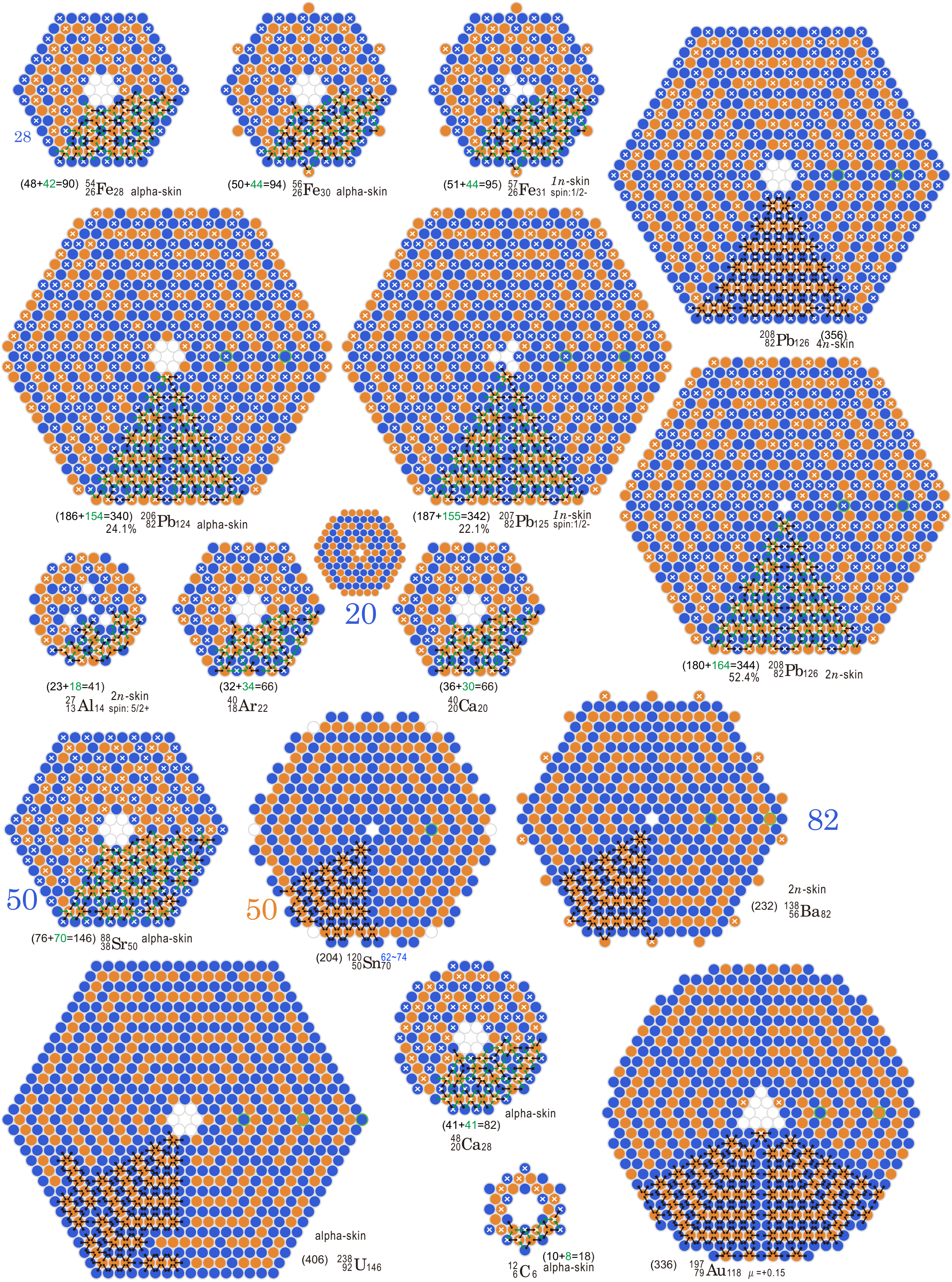


**Figure 7.** **Extended Data: $np$-SRC pair counts and nucleon arrangement across 16 representative nuclei.**
